# Malaria surveillance systems: from control to elimination

**DOI:** 10.1186/1475-2875-11-S1-O1

**Published:** 2012-10-15

**Authors:** Richard E Cibulskis

**Affiliations:** 1 Global Malaria Programme, World Health Organization, Geneva, Switzerland

## 

The capacity of malaria surveillance systems to provide accurate information on the distribution of and trends in malaria varies widely across the globe. It is influenced by (i) the extent to which patients seek treatment, (ii) whether patients use public sector health facilities, (iii) the proportion of patients that receive a diagnostic test, and (iv) the completeness of recording and reporting systems. When these factors are taken into account, it is estimated that malaria surveillance systems detect less than 10% of all cases globally, though the proportions are higher in the Europe (> 90%) and the Americas (50%). The characteristics of surveillance systems vary by geographical region. South-East Asia has the lowest percentage of malaria patients that seek treatment in public health facilities. Confirmatory diagnostic tests (blood slides or RDTs) are used infrequently in Africa, as compared with other regions. Reporting is most complete in the European region.

In April 2012 WHO released operational manuals for malaria surveillance to guide programmes both in the control phase and those in the elimination phase. In the control phase the objective of malaria programmes is to reduce the incidence of and mortality from malaria as rapidly and economically as possible. Many countries with high levels of malaria transmission are low- or lower middle-income countries, which have low expenditures per person on health care services. This results in weak health systems that are not easily accessed by the population, lower staff to patient ratios, frequent interruptions of medical supplies and limited use of parasitological diagnosis. Such settings pose particular challenges to the development of surveillance systems. Health systems in low-transmission settings are usually stronger than in high-transmission settings, and there may be widespread availability of parasitological diagnosis and appropriate treatment. Malaria may, however, be concentrated in marginalized populations, such as those living in remote border areas, migrant workers and tribal populations, and innovative ways may have to be found to reach these groups.

In the elimination phase cases occur sporadically or in distinct foci and imported cases may comprise a significant proportion of all cases. The aim of malaria programmes is to stop local transmission of malaria, and surveillance is a principal strategy for achieving this. All malaria infections are important and need to be detected, as they may lead to onward transmission (i.e., all persons with parasitaemia are considered a ‘malaria case’, regardless of the presence or absence of clinical symptoms). In practice, this is accomplished in two stages (i) by identifying all areas or *foci* with local transmission of malaria using reports of malaria cases from public and private sector health facilities. Each malaria case is then investigated to determine whether it was locally acquired or imported and, if so, from where. (ii) If a focus of local transmission is detected, the characteristics of transmission are determined and control and surveillance activities are then intensified in the focus.

